# Hydrogel Film-Immobilized *Lactobacillus brevis* RK03 for γ-Aminobutyric Acid Production

**DOI:** 10.3390/ijms18112324

**Published:** 2017-11-03

**Authors:** Yi-Huang Hsueh, Wen-Chang Liaw, Jen-Min Kuo, Chi-Shin Deng, Chien-Hui Wu

**Affiliations:** 1 Graduate School of Biotechnology and Bioengineering, Yuan Ze University, Taoyuan City 32003, Taiwan; yihhsueh@saturn.yzu.edu.tw; 2 Department of Chemical and Materials Engineering, National Yunlin University of Science and Technology, Yunlin City 64002, Taiwan; liawwc@yuntech.edu.tw (W.-C.L.); p94427p94427@gmail.com (C.-S.D.); 3 Department of Seafood Science, National Kaohsiung Marine University, Kaohsiung City 81157, Taiwan; ikuojm@webmail.nkmu.edu.tw

**Keywords:** hydrogel, hydroxyethyl methacrylate, polyethylene glycol diacrylate, gamma aminobutyric acid, immobilized, *Lactobacillus brevis*

## Abstract

Hydrogels of 2-hydroxyethyl methacrylate/polyethylene glycol diacrylate (HEMA/PEGDA) have been extensively studied for their use in biomedical and pharmaceutical applications owing to their nontoxic and highly hydrophilic characteristics. Recently, cells immobilized by HEMA/PEGDA hydrogels have also been studied for enhanced production in fermentation. Hydrogel films of HEMA/PEGDA copolymer were generated by Ultraviolet (UV)-initiated photopolymerization. The hydrogel films were used to immobilize viable *Lactobacillus brevis* RK03 cells for the bioconversion of monosodium glutamate (MSG) to γ-aminobutyric acid (GABA). The mechanical properties and fermentation yields of the *L. brevis* RK03 cells immobilized on polyacrylate hydrogel films with different monomeric formulations were investigated. Fermentation was carried out in 75 mL de Man, Rogosa and Sharpe (MRS) medium containing various concentrations of MSG. We found that HEMA (93%)/PEGDA (3%) hydrogels (sample H) maximized GABA production. The conversion rate of MSG to GABA reached a maximum value of 98.4% after 240 h. Bioconversion activity gradually declined after 420 h to 83.8% after five cycles of semi-continuous fermentation. Our results suggest that HEMA (93%)/PEGDA (3%) hydrogels have great potential for use in GABA production via semi-continuous fermentation.

## 1. Introduction

Hydrogels are composed of hydrophilic polymer networks formed by physical or chemical interactions [1]. The networks of physical hydrogels are bound together by molecular entanglements with secondary forces, while the networks of chemical hydrogels are normally crosslinked by covalent bonds [2]. Hydrogels are widely used as biomaterials due to their nontoxic nature. They are synthesized from natural polymer chains, such as collagen or alginate, or from synthetic polymers, such as poly-(vinyl alcohol) (PVA), poly(acrylic acid) (PAA), or p(2-hydroxyethyl methacrylate) (p(HEMA)). In particular, hydrogels based on p(HEMA) exhibit a degree of crosslinking and hydrophilicity similar to the properties of articular cartilage, and are often used in medical applications [3,4]. Additionally, hydrogels have great potential in other medical applications, such as drug delivery [5], regenerative medicine [6], and wound repair [7]. The literature shows that the mechanical performance of HEMA hydrogels depends on structure (especially porosity) and degree of hydrophilicity.

Chemical hydrogel synthesis by photo-crosslinking is a three-step process that involves the initiation, propagation, and termination of free radical reactions induced by UV light. First, illumination excites the photoinitiator, resulting in free radical formation; then, the radicals react with the photocurable macromonomer to produce the active substance involved in propagation. During propagation, crosslinking occurs gradually, resulting in a three dimensional (3-D) polymer network [8].

Polyethylene glycol diacrylate (PEGDA) is very hydrophilic and can suppress phase separation by increasing the solubility of the copolymer chains [4]; thus, PEGDA can be added to HEMA hydrogel formulations in different ratios to increase hydrophilicity. Furthermore, varying the ratio of HEMA to PEGDA is likely to induce noticeable changes in surface feature size and distribution. In this study, poly-hydroxyethyl methacrylate (HEMA) was modified with different ratios of polyethylene glycol diacrylate (PEGDA) to develop the in situ formation of hydrogels using UV photo-crosslinking, which were used to immobilize cells for γ-aminobutyric acid (GABA) production [3].

Cells immobilized by HEMA/PEGDA hydrogels have been studied for many years [9]. Liaw et al. (2008) fermented xylose from rice straw hemicellulose hydrolysate for xylitol production using *Candida subtropicalis* WF79 cells immobilized on polyacrylic hydrogel thin films with a thickness of 200 µm. Cell immobilization was conducted by first suspending the yeast cells in a mixture of HEMA (hydrophilic monomer), PEGDA (crosslinking agent), and benzoin isopropyl ether (photoinitiator). The maximum yield was 0.73 g of xylitol per gram of xylose consumed. In a 52.5-day-long durability test, after 40 days of repeated batchwise operation, the fermentation activity of the cells immobilized on the thin films began to decline, yielding 0.57 g/g at the end of the test. In addition, Liaw et al. (2008) prepared polyacrylate hydrogel films by combining HEMA, methacrylic acid (MAA), and *N*,*N*-dimethyl acrylamide (DMA) monomers with PEGDA (average Mw = 400 and 1000 g/mol) as the crosslinking agent. This formulation was used in the same way as an immobilization matrix for *C. subtropicalis* WF79 for the conversion of xylose to xylitol. The conversion rate reached a maximum value of 80% after 120 h, then declined after 720 h to 65%, its final value at 1080 h.

γ-Aminobutyric acid (GABA) is a non-protein amino acid, which is synthesized by the action of a pyridoxal-5′-phosphate-dependent enzyme, glutamic acid decarboxylase (GAD). GAD catalyzes the α-decarboxylation of l-glutamic acid to yield GABA [10,11]. GABA inhibits neurotransmitters in the sympathetic nervous system [12,13]; it is known to exert antidepressant [14], antihypertensive [15], and anti-diabetic effects in humans [16]. Therefore, GABA is used as a bioactive component in the pharmaceutical and food industry [17]. Various microorganisms, such as fungi [18,19], yeasts [20], and lactic acid bacteria (LAB) produce GABA [21,22,23,24,25,26,27,28,29,30,31,32,33]. Particularly, LAB have attracted attention in the food industry because they are generally recognized as safe (GRAS, an American Food and Drug Administration designation) for GABA production.

The use of immobilized cell technology in the production of GABA has been reported. For instance, embedding GABA-producing strains on calcium alginate gels for large-scale production can obtain the highest GABA biotransformation efficiency within 60 h [34,35]. Under optimal reaction conditions (pH 4.4, temperature 40 °C), GABA production reached 90%, which began to decline by the fifth consecutive production run; by the tenth production run, the production was found to be retained at 56%. Other studies revealed that bacterial cellulose membrane (BCM) vectors with ultrafine network structures were immobilized by covalently binding GAD, with a conversion rate of 87.56%. The external enzyme transformation process showed the productivity of GABA to be 6.03 g·L^−1^·h^−1^ (about 1.5 to 10.8 times more than other synthetic methods) [36].

Immobilized GAD and *L. brevis* have also been studied for many years; for instance, Lee et al. (2013) showed that His tag-mediated immobilization of *E. coli* drove glutamate decarboxylase (GAD) to convert monosodium glutamate (MSG) to GABA. This system reached a 90% conversion rate in 100 min with MSG and GAD concentrations saturated at 1.43 g/L [9]. The immobilized GAD retained 58.1% of its initial activity even after 10 consecutive cycles. Huang et al. (2007) used Ca-alginate gel beads entrapping *L. brevis* to produce GABA. The conversion rate reached approximately 90% in the first five batches (8 h per batch) and declined to 56% by the tenth batch under optimal conditions [37]. Additionally, Lee et al. (2013) showed that the immobilization of GAD in nickel-chelated sepharose reached a maximum of 97.8% conversion to GABA from 50 mM l-glutamate in a flow-through system [9].

Although the immobilization of GAD has been studied for many years, the purification of GAD is costly, and its activity decays quickly. In addition, the production of GABA by immobilization is much lower than that achieved by commercial fermentation. Therefore, it is important to find a better immobilization method for the fermentation and production of GABA. In this study, we tested different molecular weights of PEGDA (200–700 Mw) and different ratios of HEMA to PEGDA in formulations of hydrogels for bacterial cell immobilization to maximize GABA production.

## 2. Results

### 2.1. Synthesis and Characterization of Polyacrylic Hydrogel Films 

**Figure 1:**
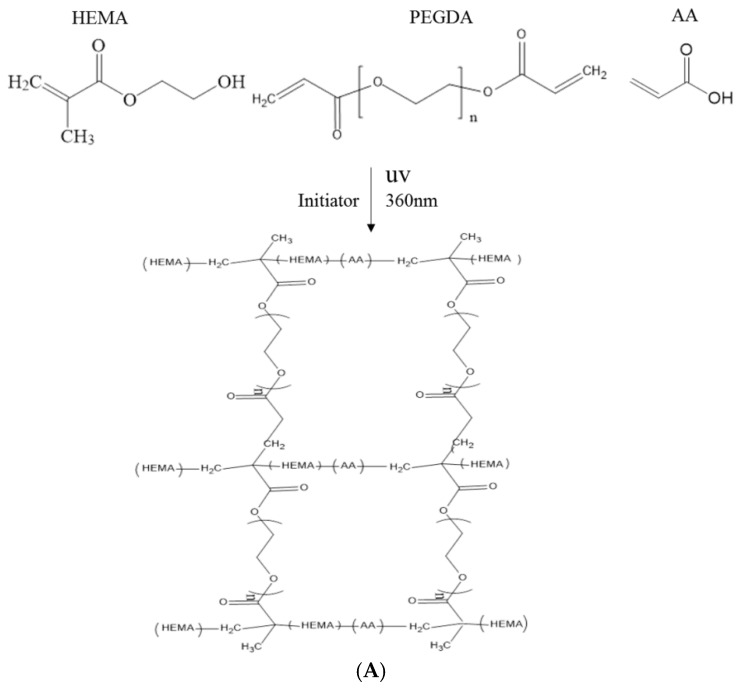

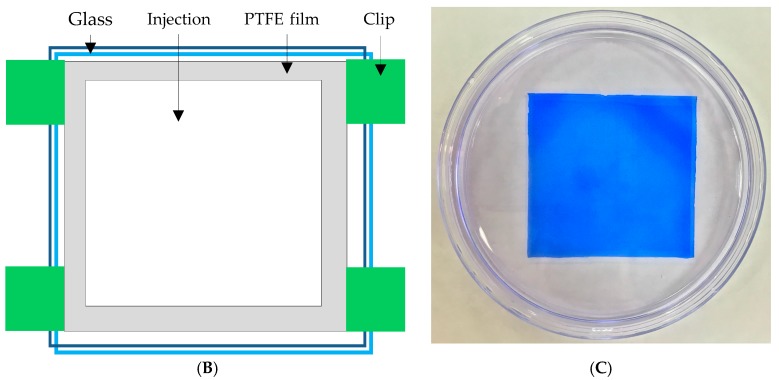
(**A**) Reaction of hydrogel synthesis using UV radiation; (**B**) Device used for hydrogel preparation; and (**C**) Photograph of the hydrogel stained with Coomassie Blue.

**Figure 2:**
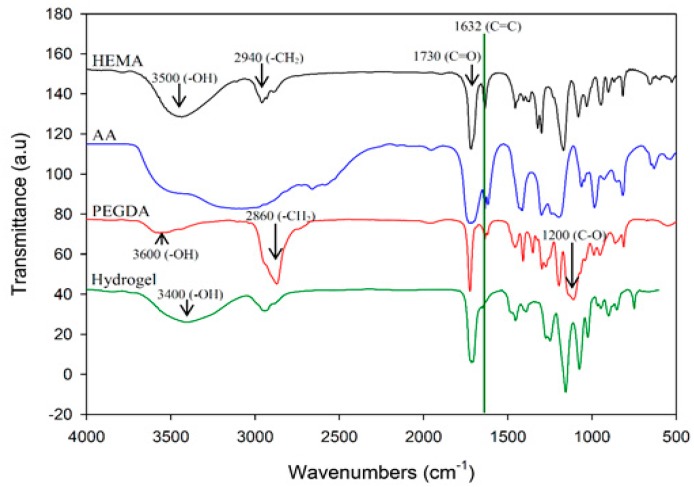
Fourier transform infrared spectroscopy (FTIR) analysis of HEMA, AA, PEGDA, and the hydrogel. Results of HEMA, AA, PEGDA, and the hydrogel are indicated in black, blue, red, and green, respectively.

In this study, polyacrylic hydrogel films were used to immobilize *L. brevis* RK03 cells for increased production of GABA. First, the polyacrylic hydrogel films were synthesized using UV radiation, as shown in Figure 1A. Different proportions of HEMA and PEGDA (Mw: 200, 400, 700) were used. Two sheets of glass with two polytetrafluoroethylene (PTFE) films were used, as shown in Figure 1B, and HEMA and PEG-DA in different proportions mixed with the photoinitiator to form the polyacrylic hydrogel films was injected, as shown in Figure 1C. To confirm the correct synthesis, FTIR spectroscopy was used to examine the HEMA, PEGDA, and hydrogel films. In the HEMA spectrum, there was an –OH bond at 3500 cm^−1^, a CH_2_ at 2940 cm^−1^, a C=O at 1730 cm^−1^, and a C=C at 1632 cm^−1^. In the PEGDA spectrum, there was an –OH at 3600 cm^−1^, a CH_2_ at 2860 cm^−1^, a C=O at 1730 cm^−1^, and a C=C at 1632 cm^−1^. In the hydrogel film spectrum, after the UV light crosslinking reaction, there was an –OH at 3400 cm^−1^, and the C=C at 1632 cm^−1^ from PEGDA had disappeared (Figure 2). This suggests that the hydrogel films were successfully synthesized after UV crosslinking. In addition, the tensile strength and water content of the hydrogels were measured. As shown in Table 1, sample A had the highest tensile strength (9.31 kg/cm^2^) but the least water content (31.41%). Sample I had the lowest tensile strength (3.95 kg/cm^2^) but the most water content (34.95%).

### 2.2. GABA Production by Immobilized Cells on Polyacrylic Hydrogel Films

**Figure 3:**
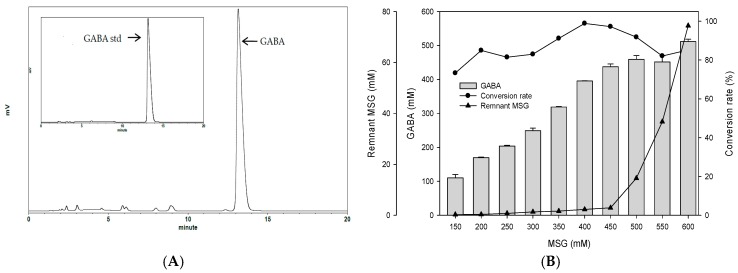

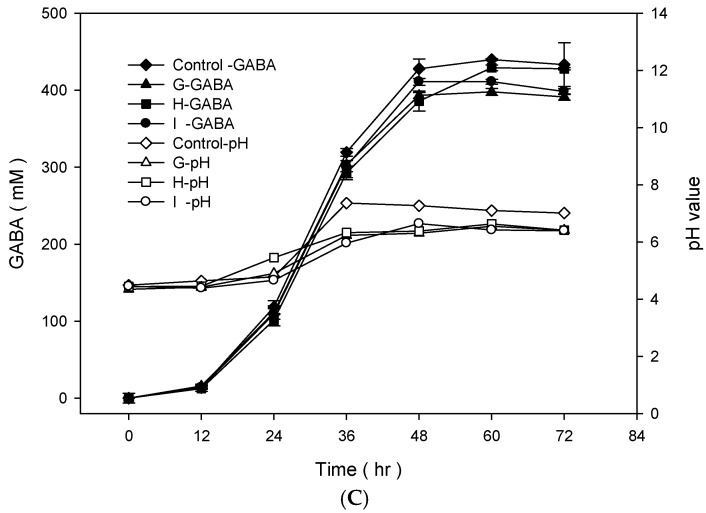
Comparison of the effect of different hydrogels on γ-aminobutyric acid (GABA) production (**A**) HPLC profiles of GABA produced by *L. brevis* RK03 grown in MRS broth with 450 mM monosodium glutamate (MSG) culture and GABA standard; (**B**) Effects of different concentrations of MSG on GABA production; (**C**) Effects of different types of hydrogels on GABA production and pH values.

**Figure 4:**
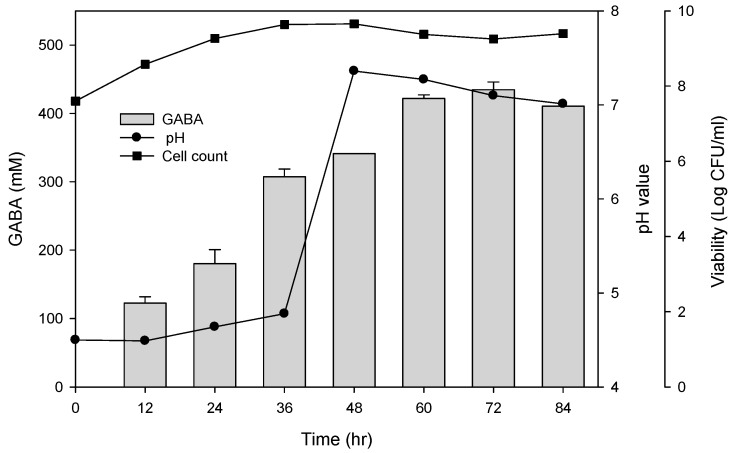
Effects of single hydrogel (sample H) biofilm formation on GABA production and pH values. (●) pH value; (■) cell count: log CFU /mL. Grey bars represent GABA concentrations.

To examine the best conditions for GABA production using cells attached to polyacrylic hydrogel films, first different MSG concentrations in de Man, Rogosa and Sharpe (MRS) medium were tested. Figure 3A shows the GABA standard and GABA production by *L. brevis* RK03 cells grown on polyacrylic hydrogel films in MRS medium with 150–600 mM MSG. HPLC analysis showed that the purified GABA matched the GABA standard. As shown in Figure 3B, the effects of MSG were compared at different concentrations added to the MRS medium, on GABA production. At 600 mM of MSG, *L. brevis* RK03 cells produced the maximum amount of GABA (511.48 mM) with a conversion rate of 85.25% in 60 h. In addition, the highest conversion rate, 98.88%, with GABA production of 395.5 mM was reached by using 400 mM MSG. However, at 450 mM MSG, cells produced higher amounts of GABA (437.64 mM) with a conversion rate of 97.25%. Therefore, 450 mM GABA as the optimal concentration was used to examine the effects of different polyacrylic hydrogel films on GABA production; we tested samples G, H, and I, which had higher water contents. Cells were grown in MRS medium with 450 mM MSG at 30 °C for 72 h. Cells grown without any polyacrylic hydrogel films and polyacrylic hydrogel film sample H had the highest GABA yield (Figure 3C), suggesting that sample H did not affect GABA production. As shown in Figure 4, *L. brevis* RK03 cells were grown in MRS medium with 450 mM MSG containing a PEGDA hydrogel film at 30 °C to investigate GABA production over 84 h. The GABA products were compared to a GABA standard, using HPLC. At 36, 48, and 60 h, the rate of GABA production increased gradually. At 36 h, the pH value was still acidic (~4.78), but at 48 h the pH had increased to 7.36. The cell density at 24 to 84 h was approximately 4.47 × 10^9^ CFU/mL. At 72 h, cells produced the maximum amount of GABA at a pH value of approximately 7.1 and at a density of 2.45 × 10^9^ CFU/mL. These results suggest that polyacrylic hydrogel films do not affect cell growth, and that maximum GABA production occurred at 72 h. In addition, the growth of biofilm cells on the hydrogels was investigated and compared to the growth of planktonic cells in MRS medium; the absorption rate was calculated as Hydrogel_cell count (log CFU/mL)_/Planktonic Control_cell count (log CFU/mL)_. The absorption rate did not differ significantly from 12 to 84 h. This suggests an increase in the numbers of planktonic cells as well as the cells growing on the hydrogel films. This also suggests that the hydrogels did not affect cell growth (Table S1).

### 2.3. GABA Production by Immobilized Cells on Polyacrylic Hydrogel Films Using Semi-Continuous Fermentation

**Figure 5:**
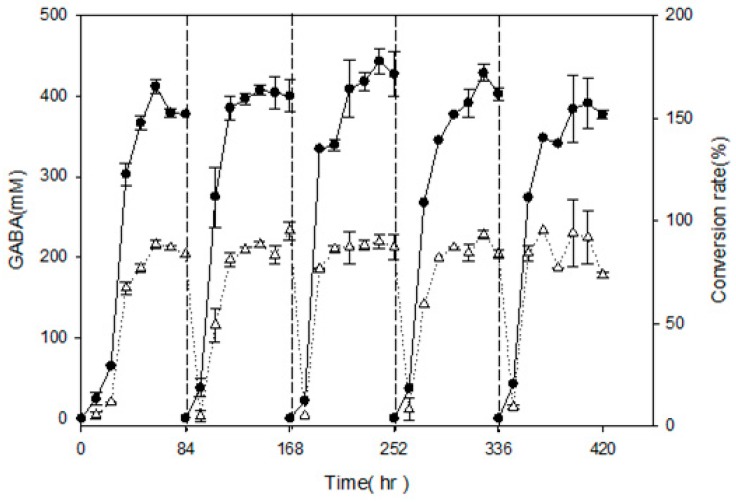
Effects of single hydrogel film adhesion on GABA production and conversion rate by *L. brevis* RK03 under semi-continuous fermentation, (●) GABA; (Δ) Conversion rate. Conversion rate of GABA from MSG (%) = amount of GABA (mM)/amount of MSG added (mM). Every 84 h, the hydrogel film was moved to fresh MRS medium for the next cycle.

In order to examine semi-continuous fermentation and maximize GABA production, *L. brevis* RK03 cells were grown on a polyacrylic hydrogel film for 84 h per cycle for a total of five cycles, and GABA production was measured every 12 h by using HPLC. After 84 h of incubation, the polyacrylic hydrogel film was moved to a new MRS medium supplemented with 450 mM MSG for another cycle; this process was repeated for four cycles. As shown in Figure 5, in every cycle except the first and second, GABA production reached a maximum at 72 h. At 60 h in the first cycle, GABA production reach its maximum at 411.86 ± 9.19 mM, and its conversion rate was approximately 91.52 ± 0.02%. In the last cycle, cells produced the least amount of GABA at approximately 384.99 ± 16.2 mM, and the conversion rate was approximately 85.33 ± 0.04%. After five cycles using polyacrylic hydrogel films to produce GABA, the conversion rate was still over 85%. This suggests that the hydrogel film system can produce over an 85% conversion rate after 17.5 days or 420 h.

### 2.4. Immobilization of L. brevis RK03 onto Polyacrylic Hydrogel Films

**Figure 6:**
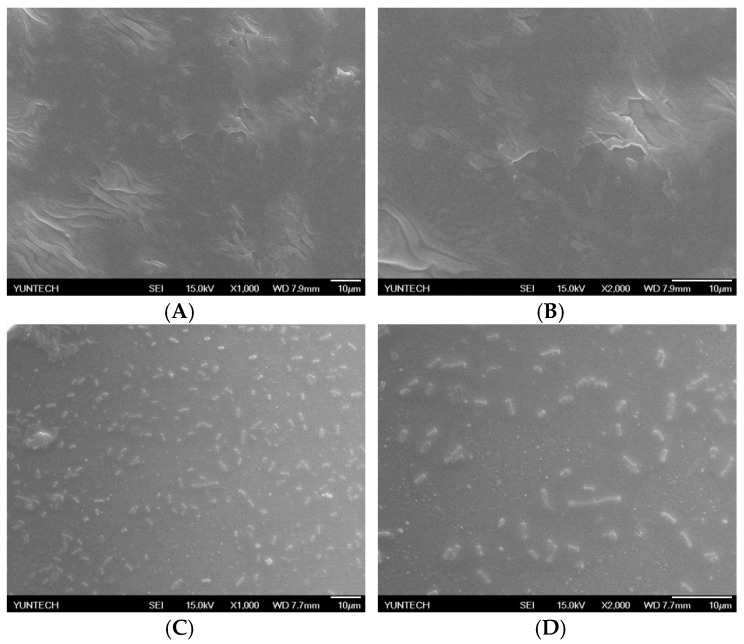

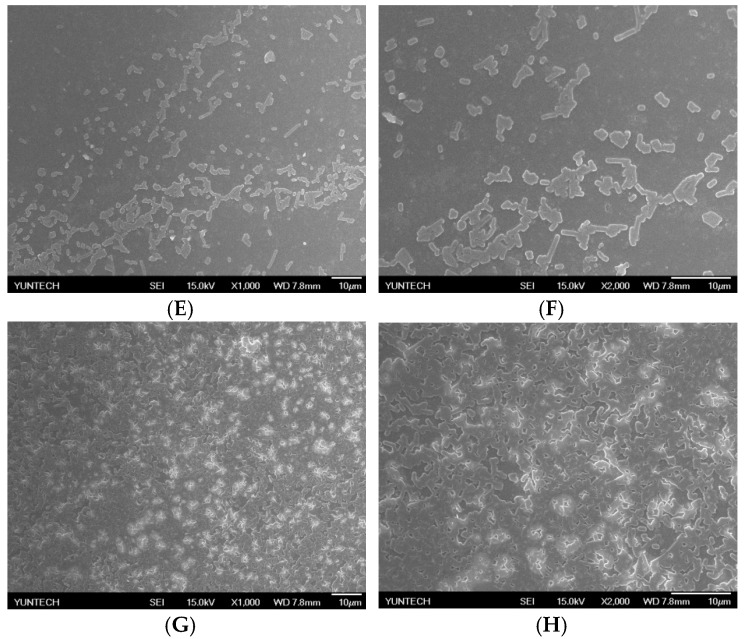
Biofilm formation by *L. brevis* RK03 on hydrogel films in MRS medium at 30 °C for 60 h. (**A**) Control 0 h, ×1000; (**B**) Control 0 h, ×2000; (**C**) 12 h, ×1000; (**D**) 12 h, ×2000; (**E**) 36 h, ×1000; (**F**) 36 h, ×2000; (**G**) 60 h, ×1000; (**H**) 60 h, ×2000.

SEM was then used to examine the *L. brevis* RK03 biofilms forming over time on polyacrylic hydrogel films. Biofilm formation peaked after 60 h (Figure 6C–H). In addition, the microstructure of the polyacrylic hydrogel films as assessed by SEM was flat with few wrinkles, as shown in Figure 6A,B (0 h). Thus, *L. brevis* RK03 was able to form biofilms on polyacrylic hydrogel films in 60 h.

## 3. Discussion

In this study, hydrogel films containing HEMA with PEGDA at a molecular weight of 700 Mw had higher water content of all formulations, including those with PEGDA molecular weights of 200 and 400 Mw. HEMA (93%) with PEGDA (3%) hydrogels (sample H) resulted in maximum GABA production by *L. brevis* RK03. This gel was reusable for up to 420 h in five 84-h cycles. Surprisingly, GABA production was 384.99 mM and the conversion rate was approximately 85%, even after five cycles of incubation. The production rate and reuse efficiency of this gel formulation are quite high and compelling.

Previously, Huang et al. (2007) embedded GABA-producing strains on calcium alginate gels and obtained the highest GABA biotransformation efficiency by 60 h; in this process, GABA conversion reached 90%, which after 10 consecutive cycles was retained at 56% [37]. Yao et al. (2013) used ultrafine network structures immobilized by covalently binding GAD; the conversion rate was found to be 87.56% and GABA productivity was 6.03 g·L^−1^·h^−1^ (approximately 1.5 to 10.8 times more than that obtained using other synthetic methods) [36]. Other researchers such as Lee et al. (2013) used immobilized GAD to convert MSG to GABA; a conversion rate of 90% was obtained in 100 min with saturated MSG and GAD at 1.43 g/L. In addition, Lee et al. (2013) showed that the immobilization of GAD on nickel-chelated Sepharose can result in a maximum conversion rate of 97.8% from 50 mM l-glutamate in a flow-through system [3]. Compared to other studies on immobilized cells or enzymes to convert MSG to GABA, our method used a hydrogel film to immobilize *L. brevis* and produce GABA. Results showed a conversion rate of 98.4%, which was retained at 83.8% after five cycles of semi-continuous fermentation. Thus, this method could produce GABA for a longer duration than that seen in previously used methods, and the conversion rate was also found to be comparatively higher. Although the immobilization of GAD has been studied for several years, its purification is costly and its enzyme activity is shown to be rapidly decayed. In addition, alginate gels that are used for immobilization to produce GABA are hard to sterilize. Therefore, we suggest that HEMA (93%)/PEGDA (3%) hydrogels have a high potential for application in semi-continuous fermentation to produce GABA, because they are easy to sterilize and can be reused. In addition, most GABA fermentation proceeds through batch fermentation but not semi-continuous or continuous fermentation. Therefore, it is important to develop a hydrogel to form biofilms in semi-continuous or continuous fermentation to increase GABA production.

## 4. Materials and Methods

### 4.1. Isolation and Identification of GABA-Producing LAB

Thirty-two LAB strains were isolated from ocean fish, including *Priacanthus macracanthus*, *Chanos*, *Perca fluviatilis*, *Thunnus thynnus*, *Psenopsis anomala*, *Ostreoida Rafinesque*, *Ephippus orbis*, *Ctenopharynodon idellus*, and *Penaeus monodon*, found at two fish markets located in the Nantze and Zuoying districts, Kaohsiung City, Taiwan. Isolated LAB strains were grown on MRS broth (BD Biosciences, Franklin Lakes, NJ, USA) plates at pH 5 supplemented with 1% monosodium glutamate (MSG) (Vedan, Taichung, Taiwan) at 37 °C. The isolated LAB strains were incubated in 9 mL MRS broth in Pyrex tubes at 37 °C without shaking for 96 h. The cell cultures containing GABA was centrifuged and filtered with 0.22-μm filters and analyzed by thin layer chromatography (TLC) assay. One pair of primers, 27F: AGA GTT TGA TCM TGG CTC AG and 1492R: CGG TTA CCT TGT TAC GAC TT, was used to amplify 16S rDNA with *L. brevis* RK03 genomic DNA as a template. The 16S rRNA gene was sequenced and aligned with genomic sequences from national center for biotechnology information (NCBI).

### 4.2. Scanning Electron Microscopy of Lactobacillus brevis RK03

The colonies were grown on polyacrylic hydrogel films in MRS medium over 60 h of incubation at 30 °C under anaerobic conditions. Scanning electron microscopy (SEM) was used to analyze the morphology of *L. brevis* RK03 cells. The cells were fixed in McDowell-Trump fixative reagent pH 7.2 (Agar Scientific Limited, Stansted, UK) for at least 2 h. The cells were washed with 0.1 M phosphate-buffered saline and centrifuged at 5000 rpm for 10 min. The resulting pellet was fixed for 1 h in 1% osmium tetroxide (Sigma-Aldrich Co., LLC, St. Louis, MO, USA) prepared in phosphate buffered saline. The sample was washed twice with distilled water for 10 min, then dehydrated for 10 min in ethanol (Merck, Darmstadt, Germany) at concentrations of 50%, 75%, 95%, and 99.5%. Afterwards, 1 mL hexamethyldisilazane (Agar Scientific Limited, Stansted, UK) was added to the sample tube for 10 min. Hexamethyldisilazane was decanted from the tube, and the cells were air-dried at room temperature. The sample specimen was coated with gold and viewed with an Hitachi Scanning Electron Microscope SU3500 (Hitachi, Tokyo, Japan).

### 4.3. Preparation of Polyacrylic Hydrogel Films for Cell Immobilization

The polyacrylic hydrogel was used to immobilize bacterial cells. First, 2-hydroxyethyl methacrylate (HEMA) and acrylic acid (AA) were mixed at different ratios, as shown in Table 1. Then, 3% of different molecular weights of polyethylene glycol diacrylate (PEGDA, Mw = 200, 400, 700 g/mol) and 1 wt % of benzoin isopropyl ether (initiator) were added (Table 1) to the above solution. The acrylic hydrogel film was prepared by injecting the above mixture into the space between two pieces of glass (50 mm × 50 mm) separated by a 0.2-mm spacer (Figure 2B,C), followed by a free-radical reaction with ultraviolet 320-nm laser excitation for 60 s. After the UV radiation reaction, the polyacrylic hydrogels were cooled and solidified with distilled water for 40 min. The synthesis procedure is shown in Figure 1A.

### 4.4. Tensile Strength and Water Content Measurement

The tensile strength of the polyacrylic hydrogel films was determined by the method specified by the Chinese National Standards (CNS) 3553, employing a dumbbell-shaped #2 sample. The rate of stretching was 2 cm/min. The tensile strength is expressed in kg/cm^2^. The polyacrylic hydrogel films were dried at 90 °C for 9 h and then weighed. Water content of the films was calculated according to the following equation: Water content = [(wet film weight − dry film weight)/wet weight] × 100%.

### 4.5. Fourier Transform Infrared Spectroscopy (FTIR) Analysis

First, the polyacrylic hydrogel films were dried at 50 °C for 24 h and the dried polyacrylic hydrogel films were then ground and mixed with potassium bromide in a 1:99 mixture and compressed into pellets. These pellets were then analyzed using FTIR (Spectrum One, PerkinElmer, Waltham, MA, USA). The monomers and polymers were analyzed on the basis of their functional groups and the wavelengths were scanned from 4000~450 cm^−1^.

### 4.6. GABA and MSG Analysis

*L. brevis* RK03 was grown in 75 mL MRS medium in 150-mL flasks supplemented with different concentrations of MSG (150–600 mM) for 72 h at 30 °C for GABA production analysis. GABA, MSG contents, and pH values were measured at certain time points. For the analysis of GABA production by *L. brevis* RK03 cells attached to different polyacrylic hydrogel films, 1 × 10^9^ CFU/mL of *L. brevis* RK03 was grown in 75 mL MRS medium supplemented with 450 mM MSG with three different polyacrylic hydrogel films (G, H, I in Table 1) for 12 h at 30 °C. Every 12 h, cell numbers, GABA content, and pH values were measured.

### 4.7. GABA and MSG Analysis in Semi-Continuous Conditions

*L. brevis* RK03 was grown in 75 mL MRS medium in 150-mL flasks supplemented with 450 mM MSG for 84 h at 30 °C for GABA production analysis. The polyacrylic hydrogel films were then removed to fresh MRS medium with 450 mM MSG for a further 84 h of incubation. The polyacrylic hydrogel films were moved this way for a further five cycles. GABA, MSG contents, and pH values were measured every 12 h.

### 4.8. Measurement of GABA Content with High-Performance Liquid Chromatography (HPLC)

A 100-μL culture broth filtered through a 0.22-μm filter was mixed with 0.5 mL *O*-phthalaldehyde (OPA) working solution (1 mL OPA stock: 40 mg *O*-phthalaldehyde/mL methanol), 25 mL of 0.1× borate buffer, and 100 μL β-mercaptoethanol) and filtered again. A GABA standard was mixed with 0.5 mL OPA working solution. Twenty microliters of the mixture was measured as follows: HPLC mobile phase A: 0.1 M sodium acetate (98%, Showa Chemical., Ltd., Tokyo, Japan) dissolved in 900 mL deionized water and 500 μL trimethylamine (Merck, Darmstadt, Germany) to a final volume of 1 L with deionized water. The pH of mobile phase A was adjusted to 6.7 using hydrochloric acid (Nihon Shiyaku Reagent, Tokyo, Japan). HPLC mobile phase B was methanol (HPLC-grade, Merck, Darmstadt, Germany). All mobile phases were passed through a 0.22-μm membrane filter. The flow rate of the pump was set at 1.0 mL/min. Column temperature was set at 30 °C, sample injection volume at 20 µL, and the compound was detected through a UV detector at 340 nm. GABA content was determined by a Hitachi 1110 pump and Hitachi 1410 detector (Hitachi High-Technologies Corporation, Tokyo, Japan) equipped with a Ascentis^®^ C18 column 5 μm in diameter, 150 mm in length, and 4.6 mm in internal diameter (Sigma-Aldrich Co., LLC, St. Louis, MO, USA). The amount of GABA was calculated by comparing the peak area with that of the GABA standard. Peak heights were measured using SISC-LAB chromatography software (Scientific Information Service Corporation, Taipei, Taiwan). To confirm the accurate detection of a GABA peak, standard curves with known concentrations of GABA were generated prior to sample analysis. Each experiment was performed in triplicate [38].

### 4.9. Statistical Analysis

Experimental data was analyzed using IBM SPSS Statistics 20 with Duncan’s multiple range test. *p*-values < 0.05 were considered statistically significant.

## 5. Conclusions

In summary, our results indicated that HEMA/PEGDA hydrogels can be used as an immobilizing material for *L. brevis* RK03 culture and GABA production for up to 420 h. Therefore, HEMA/PEGDA hydrogels can be used as a nontoxic, cheap material for the immobilization of *Lactobacillus* for use in semi-continuous or fermentative production of GABA. We are the first to demonstrate the use of this hydrogel in GABA production by *L. brevis*, and our data highlight the potential of chemically synthesized HEMA/PEGDA hydrogels as an immobilizing material in GABA production.

## Figures and Tables

**Table 1:** The tensile strength and water contents of different types of hydrogels.

No.	HEMA	PEGDA Mw: 200	PEGDA Mw: 400	PEGDA Mw: 700	AA	Initiator	Tensile Strength (kg/cm^2^)	Water Content (%)
A	95	3	-	-	1	1	9.31	31.41
B	93	3	-	-	3	1	7.26	31.56
C	91	3	-	-	5	1	6.61	32.45
D	95	-	3	-	1	1	7.52	32.10
E	93	-	3	-	3	1	6.76	32.94
F	91	-	3	-	5	1	5.96	34.63
G	95	-	-	3	1	1	7.10	34.75
H	93	-	-	3	3	1	5.20	34.91
I	91	-	-	3	5	1	3.95	34.95

HEMA: 2-hydroxyethyl methacrylate; PEGDA: Polyethylene glycol diacrylate (Mw = 200, 400, 7000 g/mol); AA: Acrylic acid; Initiator: Benzoin isopropyl ether.

## Supplementary Materials

Supplementary materials can be found at www.mdpi.com/1422-0067/18/11/2324/s1.
