# Supplementary material for: Hydrogel Film-Immobilized Lactobacillus brevis RK03 for γ-Aminobutyric Acid Production
Source: Int J Mol Sci. 2017 Nov 3;18(11):2324. doi: 10.3390/ijms18112324 (PMC5713293; doi:10.3390/ijms18112324)
Supplement: Supplementary file 1 [file ijms-18-02324-s001.pdf]

Table S1. Growth of *Lactobacillus brevis* RK03 attached onto hydrogels in MRS medium.

	Incubation Time (h)						
	**12 h**	**24 h**	**36 h**	**48 h**	**60 h**	**72 h**	**84 h**
**MRS Broth pH**	4.61	4.73	4.89	7.42	7.33	7.19	7.08
**Absorption Rate**	0.95 ± 0.14	0.98 ± 0.09	1.01 ± 0.12	1.05 ± 0.17	1.14 ± 0.11	1.03 ± 0.15	1.01 ± 0.19

Absorption rate: $Hydrogel_{\text{cell count (log CFU/mL)}}$/$Planktonic\ Control_{\text{cell count (log CFU/mL)}}$.
